# Noncoupled Mitochondrial Respiration as Therapeutic Approach for the Treatment of Metabolic Diseases: Focus on Transgenic Animal Models

**DOI:** 10.3390/ijms242216491

**Published:** 2023-11-18

**Authors:** Artem P. Gureev, Alina A. Alimova, Denis N. Silachev, Egor Y. Plotnikov

**Affiliations:** 1 Department of Genetics, Cytology and Bioengineering, Voronezh State University, 394018 Voronezh, Russia; gureev.bio.vsu@gmail.com (A.P.G.); aa10022607@gmail.com (A.A.A.); 2 Laboratory of Metagenomics and Food Biotechnology, Voronezh State University of Engineering Technologies, 394036 Voronezh, Russia; 3 A.N. Belozersky Institute of Physico-Chemical Biology, Lomonosov Moscow State University, 119234 Moscow, Russia; plotnikov@belozersky.msu.ru

**Keywords:** transgenic model, *Ciano intestinalis*, noncoupled respiration, alternative oxidase, alternative NADH dehydrogenase

## Abstract

Mitochondrial dysfunction contributes to numerous chronic diseases, and mitochondria are targets for various toxins and xenobiotics. Therefore, the development of drugs or therapeutic strategies targeting mitochondria is an important task in modern medicine. It is well known that the primary, although not the sole, function of mitochondria is ATP generation, which is achieved by coupled respiration. However, a high membrane potential can lead to uncontrolled reactive oxygen species (ROS) production and associated dysfunction. For over 50 years, scientists have been studying various synthetic uncouplers, and for more than 30 years, uncoupling proteins that are responsible for uncoupled respiration in mitochondria. Additionally, the proteins of the mitochondrial alternative respiratory pathway exist in plant mitochondria, allowing noncoupled respiration, in which electron flow is not associated with membrane potential formation. Over the past two decades, advances in genetic engineering have facilitated the creation of various cellular and animal models that simulate the effects of uncoupled and noncoupled respiration in different tissues under various disease conditions. In this review, we summarize and discuss the findings obtained from these transgenic models. We focus on the advantages and limitations of transgenic organisms, the observed physiological and biochemical changes, and the therapeutic potential of uncoupled and noncoupled respiration.

## 1. Introduction

**Figure 1:**
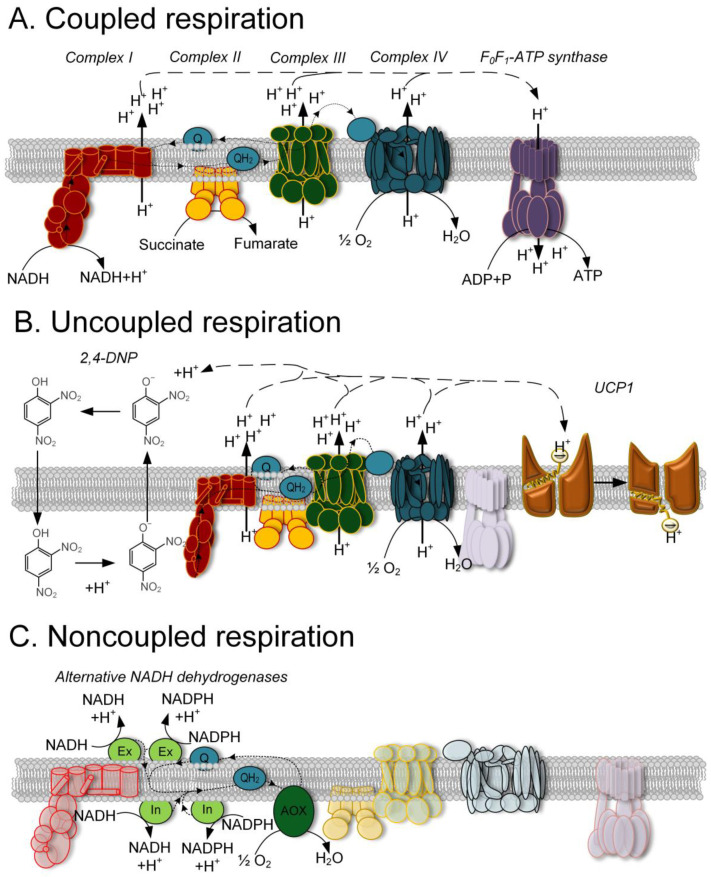
Schematic representation of the different types of mitochondrial respiration. (**A**) Coupled respiration. Electron flow from complex I and complex II through ubiquinone, cytochrome c complex III, and complex IV results in the transport of H^+^ from the mitochondrial matrix into the intermembrane space, generating a membrane potential. The membrane potential is then used for ATP synthesis by F_0_F_1_-ATP synthase. (**B**) Uncoupled respiration. Reverse leakage of H^+^ across the membrane occurs either through chemical uncouplers (represented by the classical example of 2,4-dinitrophenol) or through protein uncouplers (represented by UCP1). (**C**) Noncoupled respiration. This is characteristic of plants and certain sessile animals. There is no generation of a membrane potential because NAD(P)H oxidation is carried out by internal and external NADH dehydrogenases. Alternative oxidase (AOX) serves as a terminal complex utilizing oxygen, and electron flow through these proteins is not coupled to the transport of H^+^ across the inner mitochondrial membrane.

Mitochondrial respiration can be divided into three types according to its ability to form a membrane potential (∆μH^+^). Energy-coupled respiration allows the generation of ∆μH^+^ and it is utilized for ATP synthesis (Figure 1A). During uncoupled respiration, ∆μH*^+^* is formed but immediately dissipated without ATP synthesis by specific proteins or in the presence of certain substances called uncouplers (Figure 1B). During noncoupled respiration, electron flow is not associated with ∆μH^+^ formation (Figure 1C) [1]. Energy-coupled respiration is facilitated by protein complexes that form the respiratory chain. The energy carried by electrons flowing through this electron transport chain is used to transport protons across the inner mitochondrial membrane. This generates potential energy in the form of an electrochemical gradient on the inner mitochondrial membrane. Subsequently, using this potential, F_0_F_1_-ATP synthase generates ATP from ADP and inorganic phosphate (Figure 1A) [2]. While oxidative phosphorylation is an extremely efficient process, a significant portion of the electron energy can be diverted towards the synthesis of free radicals, which generally have a negative impact on the structural integrity of membranes, proteins, and DNA [3]. For this reason, uncoupled and noncoupled respiration plays an important role in maintaining cellular redox homeostasis. 

The physiological role of uncoupled respiration is multifaceted. Uncoupled respiration, primarily mediated by proteins from the UCP family, has been extensively studied. UCP1, found in brown adipose tissue, plays a crucial role in non-shivering adaptive thermogenesis. Through proton leak across the mitochondrial inner membrane, UCP1 dissipates the ∆μH^+^, leading to increased substrate oxidation and heat production independently of ATP synthesis [4]. The activation of UCP1 is facilitated by long-chain fatty acids (LCFAs). Initially, the LCFA anion binds to UCP1 on the cytosolic side of the membrane. Subsequently, H^+^ binds to the LCFA, triggering a conformational change that releases H+ on the opposite side of the inner mitochondrial membrane. The LCFA anion remains associated with UCP1 through hydrophobic interactions established by its carbon tail. The LCFA anion then returns for another cycle of H^+^ translocation (Figure 1B) [5]. Another mechanism of uncoupling oxidative phosphorylation involves specific small molecules that participate in proton translocation from the intermembrane space to the mitochondrial matrix. Protonophores are classic uncouplers that can directly transport protons across the inner mitochondrial membrane through their redox properties and lipophilic structure (Figure 1B) [6]. Non-protonophore uncouplers, on the other hand, typically act as agonists of other proteins which are involved in the regulation of ∆μH^+^ [7]. 

Noncoupled respiration is energetically similar to uncoupled respiration but has a different origin. Noncoupled respiration is facilitated by special respiratory complexes that participate in electron transport without forming ∆μH^+^. Classic examples of noncoupled respiration are found in alternative respiratory pathways in plant mitochondria [8]. Plant mitochondria contain at least five additional components in the electron transport chain. Four of these components catalyze the transfer of NADH or NADPH to ubiquinone, while the fifth component is an alternative oxidase (AOX) that directly catalyzes the transfer of electrons to molecular oxygen [9,10]. Alternative respiratory pathways do not involve the transport of protons across the inner mitochondrial membrane and, therefore, are not coupled with ATP synthesis (Figure 1C). With a few exceptions, animals, due to their active lifestyles, generally lack these alternative respiratory pathways [11]. Hence, animal mitochondria are highly susceptible to various poisons and xenobiotics that can inhibit electron flow and cause electron “leaks”, leading to the excessive production of reactive oxygen species (ROS) [12]. Mutations in mitochondrial DNA and nuclear DNA genes associated with the respiratory chain can also result in severe metabolic defects and pathologies [13]. 

**Figure 2:**
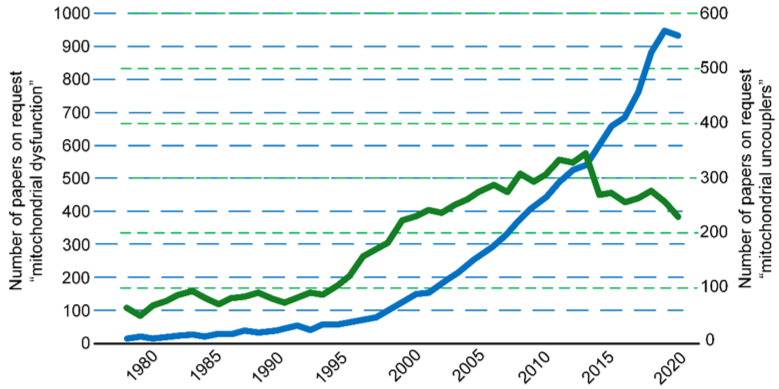
Green line indicates the number of publications in PubMed database related to “mitochondrial uncouplers” (https://pubmed.ncbi.nlm.nih.gov/) (accessed 22 July 2023). It is noticeable that the number of publications dealing with mitochondrial uncouplers has decreased over the last decade. In contrast, interest in mitochondrial dysfunctions, represented by the blue line indicating the number of publications in the PubMed database on the topic of “mitochondrial dysfunctions”, is growing exponentially.

Thus, the main, but not the only, positive effect of uncoupled and noncoupled respiration is the normalization of the redox state to reduce the production of free radicals, which is expected to significantly reduce the progression of various diseases [14]. In general, chemical uncouplers show promise as pharmacological agents for treating various metabolic disorders. However, it should be acknowledged that in recent years, there has been a waning of interest in chemical uncouplers, despite the growing interest in the investigation of mitochondrial pathologies (Figure 2). This is likely due to the fact that many classical and well-studied uncouplers have a very narrow therapeutic window. Perhaps this is because some uncouplers have been “discredited” due to a high number of side effects [15,16]. Therefore, while this review briefly addresses the topic of chemical uncouplers, its primary focus is on the comprehensive discussion of transgenic animal models pertaining to uncoupled and noncoupled respiration. Undoubtedly, genome editing as a therapeutic approach for human diseases is not yet considered a viable method. However, transgenic animal models allow for a better assessment of the effects of uncoupled and noncoupled respiration on cellular, tissue, and organismal states. These effects can be evaluated at different stages of organism development (from embryonic to aging stages) and under conditions of various induced diseases. A deeper understanding of these processes will help in developing new therapeutic approaches for treating metabolic disorders by regulating the degree of coupling in mitochondrial respiration. 

## 2. Chemical Uncouplers

Mitochondrial uncouplers are synthetic compounds that belong to various classes of chemicals. These uncouplers exhibit multiple mechanisms of action, enabling them to be classified indirectly only. Based on their functionality, uncouplers can be divided into those that exhibit a protonophore effect, while others indirectly induce uncoupling by regulating the activity of uncoupling proteins or altering mitochondrial function [17]. Protonophores are typically hydrophobic aromatic compounds with a negative charge. These compounds have the ability to distribute negative charge among multiple atoms through π-orbitals, thus facilitating the delocalization of a proton’s charge upon its attachment to the molecule [18]. It allows them to easily penetrate through the lipid membrane and move in their neutral form along the concentration gradient. In addition to this, protonophores can interact with proteins within the inner membrane [6].

2,4-Dinitrophenol (2,4-DNP) is commonly considered a classical protonophore. As early as 1933, it was discovered that the use of 2,4-DNP leads to rapid weight loss by enhancing the basal metabolism [19], resulting in the accelerated metabolism of fats and carbohydrates [20]. However, at that time, the concept of protonophores and the mechanism of 2,4-DNP action were not yet established. By 1938, the sale of 2,4-DNP without a prescription was prohibited, and shortly thereafter, it was completely banned [21]. The rapid weight loss in patients was accompanied by side effects attributable to specific metabolic characteristics. These included the shifting of the electrochemical gradient and dissipation of potential energy as heat, leading to uncontrolled hyperthermia [22], the inhibition of mitochondrial inorganic phosphate uptake [23], the excessive stimulation of glycolysis [24], and the accumulation of Na^+^ and K^+^ [25,26] (Supplementary Table).

The term protonophore was introduced by Skulachev in 1970 [27]. By this time, other protonophores from the hydrazone class, carbonyl cyanide-p-trifluoromethoxyphenyl hydrazone (FCCP) and carbonyl cyanide m-chlorophenyl hydrazone (CCCP), had already been discovered [28]. However, they also exhibit non-specific effects on other organelles, including the cytoplasmic membrane [29]. Their effects can induce both the depolarization and hyperpolarization of the cytoplasmic membrane by influencing H^+^, Na^+^, K^+^, and Ca^2+^ channels [30,31]. Among other well-studied protonophores, it is worth mentioning FR58P1 [32], BAM15 [33], C12TPP [32], C12R1 [34], CDE [35], C4R1 [36], bupivacaine [37], catechin [38], fisetin [38], quercetin [38], apigenin [38], usnic acid [39], and others. Other compounds can induce uncoupling effects by targeting the mitochondrial membrane protein PTEN-induced kinase 1 (PINK1) (niclosamide [40], triclosan [41], sertraline [41]; the metabolic regulator AMP activated protein kinase (AMPK) (curcumin [42], sorafenib [43], SR4 [44], FR58P1a [32], FH535 [45]), as well as the uncoupling proteins of the UCP family (T3 [46]) (see Supplementary Materials). In addition, some antioxidants have an uncoupling effect, which makes them more promising for further use by compounds [47]. Some compounds are derivatives of previously studied substances, developed with the aim of enhancing their therapeutic properties and reducing toxicity (niclosamide piperazine [48] and DNPME [49]) (Supplementary Materials).

Chemical mitochondrial uncouplers are actively investigated in scientific research for the development of new approaches in the treatment of neurodegenerative diseases such as Alzheimer’s disease [50], Parkinson’s disease, traumatic brain injury and stroke [36,51], ischemic heart disease [52,53], liver diseases [54], kidney diseases [33], as well as various forms of obesity [55,56], diabetes [54], and cancer [57]. In the middle of the 20th century, niclosamide was used as an antihelmintic drug. But then, its other properties were discovered [58] (see Supplementary Materials). However, it should be noted that their use for medical purposes requires further research and testing (Supplementary Materials). 

This chapter provides only brief information about the nature and medical applications of synthetic uncouplers, as there are already numerous reviews dedicated to this topic. The focus of the current review will now shift towards transgenic animal and cellular models that simulate intense uncoupled and noncoupled respiration without pharmacological intervention.

## 3. Transgenic Models Which Overexpress UCPs

**Figure 3:**
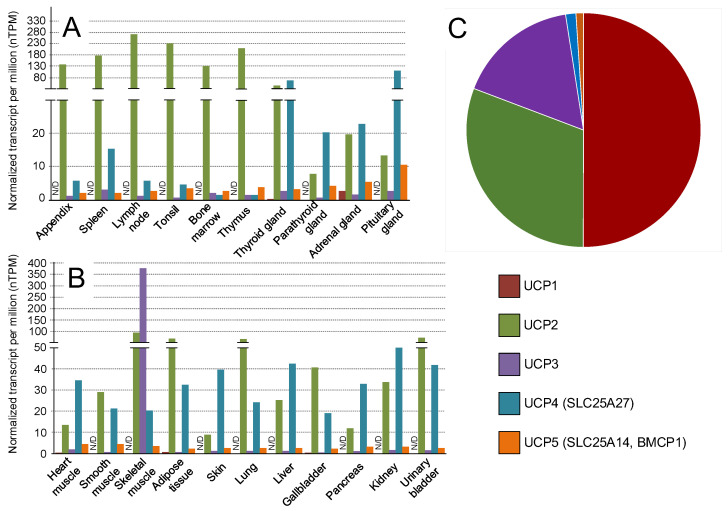
Normalized expression of UCP1-5 genes in the endocrine tissues and lymphoid system (**A**) and in different organs (**B**). Data were collected from the Human Protein Atlas (https://www.proteinatlas.org/) (accessed 22 July 2023). The number of publications for each query, “UCP1”, “UCP2”, “UCP3”, “UCP4”, “UCP5”, presented in the PubMed database (https://pubmed.ncbi.nlm.nih.gov/) (accessed 22 July 2023) (**C**).

The most well-characterized and studied member of the UCP gene family is UCP1 (Figure 3C). However, adult humans exhibit minimal UCP1 expression. It is specifically expressed in brown adipose tissue, which is abundant in newborns and infants during early childhood. For a long time, it was believed that brown adipose tissue was absent in adults [59]. However, at the beginning of the 21st century, it was discovered that accumulations of brown adipose tissue, larger than 4 mm in diameter, are present in 7.5% of women and 3.1% of men. There are cervical, supraclavicular, and upper mediastinal depots of brown adipose tissue. [60]. The relatively high expression of UCP1 is also observed in the adrenal gland [61] (Figure 3A). UCP1 is expressed to a lesser extent in white adipose tissue (Figure 3B).

Various studies have shown that UCP1 is mainly expressed in the back subcutaneous adipose tissue, perirenal adipose tissue, or visceral adipose tissue [62]. In inbred Lou/C rats, which are a transgenic model of obesity resistance, increased *Ucp1* expression was observed in white adipose tissue, potentially contributing to resistance to diet-induced obesity [63]. A transgenic model that expresses *Ucp1* in gastrocnemius muscle (MCK-UCP1-20) showed lower body weight and specifically decreased muscle mass despite consuming the same amount of food. Importantly, no cardiac muscle pathology was found in the MCK-UCP1-13 mouse strain, which also expressed Ucp1 [64]. Moreover, the MCK-UCP1-13 mice exhibited improved functional recovery after heart ischemia/reperfusion [65]. Another transgenic model, HSA-mUCP1, with increased Ucp1 expression in the muscles, exhibited increased respiratory quotient levels, indicating overall increased glucose oxidation [66]. Additionally, the expression of Ucp1 in skeletal muscle reduced the risk of reverse electron transfer in the mitochondrial respiratory chain and ROS production [67]. It is known that reverse electron transport to complex I of the respiratory chain is one of the factors contributing to the ROS hyperproduction, particularly in in vitro mitochondrial systems [68]. The impact of *Ucp1* expression was also studied in kidney injury models, in which the viral-based overexpression of *UCP1* reduced the mitochondrial ROS generation and apoptosis in hypoxia-treated tubular epithelial cells [69] (Table 1). 

**Figure 4:**
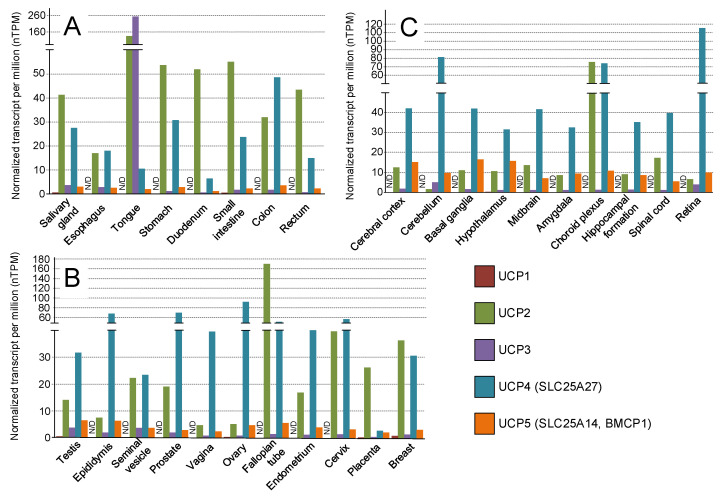
Normalized expression of UCP1-5 genes in the gastrointestinal tract (**A**), in the male and female reproductive systems (**B**), in the different brain compartments (**C**). Data were obtained from the Human Protein Atlas (https://www.proteinatlas.org/) (accessed 22 July 2023).

UCP2 is a well-studied uncoupling protein (Figure 3C) that is mainly expressed in organs and cells associated with the immune system [85] (Figure 3A). The analysis of its expression patterns also shows high expression levels in almost all sections of the digestive system, adipose tissue, smooth muscle, lungs, and gallbladder (Figure 3B and Figure 4A). UCP2 is expressed at a lower level (compared to UCP4) in the brain (Figure 4C), predominantly in axons and axon terminals. The heat generated by axonal UCP2 modulates neurotransmission in homeostatic centers, coordinating the activity of brain circuits that regulate energy balance and related autonomic and endocrine processes [86]. Drosophila models overexpressing human *hUCP2* in the nervous system have shown increased lifespan, reduced oxidative damage, and enhanced resistance to paraquat and rotenone, both of which are commonly used to create Parkinson’s disease models [70,71]. Similarly, transgenic mice overexpressing *Ucp2* in catecholaminergic neurons exhibited similar effects. These mice showed a twofold elevation in *Ucp2* expression in dopaminergic neurons of the substantia nigra, resulting in increased mitochondrial uncoupling. When acutely exposed to 1-methyl-4-phenyl-1,2,3,6-tetrahydropyridine (MPTP), a neurotoxin used in Parkinson’s disease models, transgenic mice demonstrated neuroprotection and retained locomotor functions [60] (Table 1).

The impact of *Ucp2* overexpression has been studied in various neurological disease models. Contradictory results have been obtained in amyotrophic lateral sclerosis (ALS) models. On the one hand, it has been shown that the overexpression of hUCP2 increased the survival age of superoxide dismutase 2 knockdown (sod2^−/−^) mice and reduced ROS production and oxidative stress throughout the aging process [72]. Conversely, other studies conducted on the same transgenic model showed that hUCP2 overexpression worsens mitochondrial dysfunction and accelerates ALS progression [73]. The positive effects of UCP2 overexpression have been consistently observed in ischemic disease models. The overexpression of *Ucp2* protected thalamic neurons following global ischemia [74] and attenuated the increase in IL-6 levels and decrease in Bcl2 levels following focal ischemia [75]. *Ucp2*-overexpressing mice demonstrated faster recovery rates after middle cerebral artery occlusion (MCAO)-induced stroke and traumatic brain injury [76]. Rat transgenic models overexpressing *Ucp2* have not been created. However, the injection of a lentiviral vector encoding UCP2 (LV-UCP2) into stroke-prone spontaneously hypertensive rats (SHRSP), fed with a high-salt Japanese-style diet, resulted in the delayed onset of stroke and kidney injury [87]. In transgenic mice constitutively expressing *Ucp2* in the hippocampus prior to epileptic seizure induction, a substantial reduction in cell death was observed [78]. *Ucp2* expression in transgenic animals decreased retinal ganglion cell degeneration and death in a mouse model of glaucoma [77]. It is worth noting that the overexpression of UCP2 has a therapeutic effect not only in neurological and neurodegenerative diseases. It has been shown that the targeted expression of *Ucp2* in mouse liver increases susceptibility to acute liver injury induced by lipopolysaccharide and galactosamine [79] (Table 1).

UCP3 is primarily expressed in skeletal muscles [88] (Figure 3B). The data from the Protein Atlas indicates that the high expression of UCP3 is observed in the tongue, which is not contradictory to the previous statement, as the tongue is a muscle organ (Figure 4A). Mice overexpressing human UCP3 in skeletal muscle (UCP-3tg) exhibited hyperphagia along with a significant reduction in adipose tissue mass [80] and increased β-oxidation through a thioesterase-1-dependent mechanism in the mitochondria [81]. However, the overexpression of Ucp3 in the skeletal muscle of transgenic mice was also accompanied by an increase in muscle mitochondrial inefficiency, as indicated by a reduction in the ratio of ATP synthesis to mitochondrial oxidation [82] (Table 1).

UCP4, or solute carrier family 25 member 27 (SLC25A27), is primarily expressed in the brain and in male and female reproductive systems (Figure 4B). However, it plays a lesser role in uncoupling oxidative phosphorylation, as it is preferentially localized in close vicinity to VDAC, presumably at the inner boundary membrane of neuronal mitochondria, whereas F_0_F_1_-ATP synthase is more centrally located at the cristae membrane. Therefore, due to the distinctive mitochondrial morphology, UCP4 is unlikely to function as a direct uncoupler of oxidative phosphorylation. However, this observation supports the possibility that UCP4 may instead play a role in dissipating the excessive proton gradient typically linked to ROS production [89]. No information was found regarding transgenic models that overexpress UCP4. However, targeted overexpression was induced using lentiviruses and vectors. The viral-induced overexpression of UCP4 improved neuronal survival in vitro in a mouse model of Alzheimer’s disease and prevented spatial memory impairments in vivo in 3xTg mice [90]. The lentiviral-induced overexpression of UCP4 in astrocytes was found to promote neuronal survival. The reduction in ATP production was effectively compensated by an enhancement of glycolysis, which resulted in nonoxidative energy production without deleterious H_2_O_2_ generation. It was observed that astrocytes exhibiting higher levels of UCP4 produced increased amounts of lactate, which served as an energy source for neurons and facilitated enhanced neuronal survival [91].

UCP5 (also known as BMCP1, brain mitochondrial carrier protein-1) is similarly expressed predominantly in the nervous system (Figure 4C), like UCP4. It is the least studied member of the UCP family (Figure 3C), but cellular cultures overexpressing UCP5 have been obtained. Neuronal (GT1-1) cell lines with the stable overexpression of UCP5 showed a lower mitochondrial ∆μH^+^, indicating the stronger uncoupling of mitochondria, as well as reduced ATP production [83]. The stable overexpression of UCP5 provided protection against 1-methyl-4-phenylpyridinium (MPP*^(+)^*)- and dopamine-induced toxicity in SH-SY5Y neuroblastoma cells [84] (Table 1).

## 4. Transgenic Animal Models in Which Components of Alternative Respiratory Pathways Are Expressed

The discovery of gene-encoded proteins for alternative respiratory pathways in the genomes of some animals in 2004 has provided important background for the future development of transgenic animal models [11]. Ciona intestinalis, an ascidian, has been widely used as a donor for the alternative oxidase (AOX) in transgenic models in numerous studies [92,93,94,95,96,97,98,99]. Additionally, in two studies, C. intestinalis was utilized as the source of alternative NADH dehydrogenases [100,101]. Saccharomyces cerevisiae has been the primary source of alternative NADH dehydrogenases in most studies [102,103,104,105]. There has also been reported a study in which alternative NADH dehydrogenases from plants were transfected into human cells [106] (Table 2).

There are two main therapeutic effects resulting from the expression of alternative respiratory pathways in insect and mammalian cells. The first effect is associated with the restoration of the respiratory rate following inhibition or damage to the subunits of respiratory chain complexes. The expression of alternative NADH dehydrogenases restored the respiration rate in cells with defective complex I cells [106], in flies with the reduced expression of the complex I assembly factor [102], as well as during complex I inhibition by rotenone [101,104,105] and paraquat [105]. The expression of AOX led to the restoration of the respiratory rate upon the inhibition of complex III by antimycin [99], complex IV by cyanide [93,97,99], azide [99], cigarette smoke condensate [96], mutations in genes encoding complex IV subunits [92,95,107], and gene knockdown responsible for complex IV assembly [95] as well as in complex III-deficient mice [98].

The second effect is related to the modulation of ROS metabolism. The expression of alternative NADH dehydrogenases induced a decrease in the rate of ROS production [102,104,105] and suppressed the levels of oxidative stress markers [102,105,106]. AOX expression reduced the rate of ROS production [95,99], levels of oxidative stress markers [94], and the rate of superoxide production induced by cyanide [93], antimycin [92,93,97], menadione [100], and cigarette smoke condensate [96].

The therapeutic potential of alternative respiratory pathways has been demonstrated in models of Alzheimer’s disease [94], Parkinson’s disease [95], Leigh syndrome [95], and cardiomyopathy [92,98]. It has been shown that AOX expression may be associated with the activation of signaling pathways linked to cell survival and protection against oxidative stress, particularly the Nrf2/ARE signaling pathway [101].

However, it would be incorrect to assume that the expression of genes encoding components of alternative respiratory pathways is capable of resolving all mitochondrial dysfunctions. The tko25t mutant Drosophila, which carries a recessive point mutation in the gene for mitoribosomal protein S12, demonstrates a decreased abundance of mitoribosomal small subunits, multiple respiratory chain dysfunctions, and ATP synthase deficiency [108]. The expression of AOX from C. intestinalis does not rescue the tko25t phenotype. Additionally, the expression of Ndi1 by S. cerevisiae during development is lethal for tko25t [109]. Moreover, the expression of Ndi1 exacerbates the neuronal phenotype resulting from complex IV subunit knockdown [107]. The overexpression of monocyte chemoattractant protein 1 (Mcp1) in mice cardiomyocytes induces inflammatory cardiomyopathy, leading to death from heart failure at the age of 7–8 months. AOX is unable to rescue heart failure directly caused by complex IV deficiency in mice overexpressing Mcp1 [110]. Concerns have been raised that a drastic decrease in the rate of superoxide production, dependent on AOX, may impair the functioning of signaling pathways associated with ROS metabolism [93]. Catania et al. (2019) showed that the affinity of alternative NADH dehydrogenase from Arabidopsis thaliana to NADH is over 3-fold higher than the affinity of complex I for NADH in human fibroblasts. This could potentially have a negative impact on ATP production and the metabolic status of the entire organism [106].

## 5. Conclusions

With rare exceptions, we observe that transgenic models simulating uncoupled and noncoupled respiration have shown positive effects in various disease models. However, it is important to recognize that implementing this approach as a therapeutic strategy is currently challenging and not yet practical in clinical practice. In the context of uncoupled respiration, synthetic uncouplers serve as an alternative for transgenic models that theoretically could be implemented in clinical practice not only for eliminating helminths [58] but also for treating metabolic and neurodegenerative diseases in humans. However, the situation is more difficult when it comes to the analogs of noncoupled respiration, which is characteristic of plants and certain types of sessile animals. Currently, we are not aware of any specific compounds that prevent the formation of a membrane potential without inhibiting any respiratory complexes. However, in our opinion, the closest model for simulating noncoupled respiration is provided by methylene blue, which facilitates alternative electron transport [111]. Methylene blue can accept electrons from NADH, the succinate dehydrogenase complex, and the alpha-glycerophosphate dehydrogenase complex, and then transfer them to cytochrome c [112]. Methylene blue can act as a bypass for an inhibited or damaged complex I [113], similar to how plant alternative NADH dehydrogenases function. As a result, when using methylene blue, electrons from the reducing equivalents pass through fewer coupling complexes compared to fully coupled respiration. Today, the intravenous injection of methylene blue is approved by the Food and Drug Administration (FDA) (Accession Number: DB09241) and European Medicines Agency (EMA) (Agency product number: EMEA/H/C/002108) for the treatment of patients with acquired methemoglobinemia. Clinical trials are underway for its potential therapeutic use in Alzheimer’s disease (Accession Number: NCT03446001). Studies suggest that methylene blue may slow the progression of Parkinson’s disease [114], Huntington’s disease [115], amyotrophic lateral sclerosis [116], and cognitive decline associated with aging [117,118,119,120,121,122,123,124,125,126,127,128,129,130,131,132,133,134,135,136,137,138]. Consequently, targeting the mitochondrial respiratory chain to reduce the tension on the inner mitochondrial membrane or bypass inhibited or damaged respiratory complexes represents a promising direction that requires further investigation.

## Figures and Tables

**Table 1:** Transgenic models in which UCPs are overexpressed.

Overexpressed Gene	Transgenic Strains	Model of Disease	Effect	Reference
UCP1	Transgenic strains expressing UCP1 in muscle and heart		Body weight was reduced with the same food intake. The decrease in weight mainly occurred in muscle tissue. No changes were observed in cardiac muscle	[64]
UCP1	Transgenic strains expressing UCP1 in muscle and heart	Heart ischemia/reperfusion	Functional recovery on reperfusion was improved	[65]
UCP1	HSA-mUCP1 mice expressing UCP1 in the skeletal muscles		RQ level was increased, indicating an overall increase in glucose oxidation	[66]
UCP1	HSA-mUCP1 mice expressing UCP1 in the skeletal muscles		UCP1 expression in skeletal muscle reduced the risk of reverse electron transfer and the production of reactive oxygen species	[67]
UCP1	Lou/C rats with UCP1 overexpression in WAT	Obesity	Prevented body weight gain, decreased fat mass, and improved insulin sensitivity	[63]
UCP2	Transgenic fly, *UAS-hUCP2*		Increased hUCP2 expression in the adult nervous system, extended life span	[70]
UCP2	Transgenic fly, *UAS-hUCP2*	PD model	Less ROS accumulation, heightened resistance to rotenone-induced lethality, and extended life span	[71]
UCP2	Transgenic mice overexpressing UCP2 in catecholaminergic neurons (TH-UCP2)	PD model	Upon acute exposure to MPTP, TH-UCP2 mice showed neuroprotection and retention of locomotor functions	[60]
UCP2	Transgenic mice overexpressing hUCP2	ALS model	Increased survival of *sod2*^−/−^ mice	[72]
UCP2	Transgenic mice overexpressing hUCP2	ALS model	Worsened mitochondrial dysfunction and accelerated disease progression of *sod2*^−/−^ mice	[73]
UCP2	UCP2/3 transgenic overexpressing mice	Global ischemia	Overexpression of UCP2 protects thalamic neurons following global ischemia	[74]
UCP2	UCP2/3 transgenic overexpressing mice	Focal ischemia	Overexpression of UCP2 blunted the ischemia-induced increase in IL-6 and decrease in Bcl2.	[75]
UCP2	UCP2/3 transgenic overexpressing mice	Stroke TBI	Overexpression of UCP2 enhased of neurological recovery	[76]
UCP2	Ucp2KI^fl/fl^ mice	Glaucoma	Decreased glaucomatous cell death	[77]
UCP2	UCP2/3-overexpressing mice	Epileptic seizures	Increased mitochondrial number and ATP levels with a parallel decrease in free radical-induced damage	[78]
UCP2	Transgenic mice with targeted expression of UCP2 in the liver	Acute liver injury	Expression of UCP2 in mouse liver increases susceptibility to acute liver injury induced by lipopolysaccharide and galactosamine	[79]
UCP3	Mice overexpressing human UCP-3 in skeletal muscle (UCP-3tg)		Mice were hyperphagic but weighed less	[80]
UCP3	Mice overexpressing human UCP-3 in skeletal muscle (UCP-3tg)		UCP-3tg showed increase in β-oxidation in the MTE-1-dependent manner	[81]
UCP3	Mice overexpressing human UCP-3 in skeletal muscle (UCP-3tg)		UCP-3tg showed increase in muscle mitochondrial inefficiency and decrease in ATP synthesis	[82]
UCP5	UCP5-transfected cell lines of heart and kidney		The mitochondrial ROS production was decreased	[83]
UCP5	SH-SY5Y neuroblastoma cells stably overexpressing human UCP5	PD model	UCP5 overexpression protected against MPP^(+)^- and dopamine-induced toxicity	[84]

**Table 2:** List of transgenic models in which component of alternative respiratory pathways was expressed.

Model Object	Gene	Donor Organism	Complex Bypass	ROS Effect	Model of Disease	Reference
Cell lines						
Flp-In^TM^ T-REx^TM^-293 cells	AOX	*C. intestinalis*	CIV dysfunction. Cyanide-induced inhibition	Decrease in antimycin-induced superoxide overproduction		[97]
COX10-depleted HEK-293-derived AOX-transgenic cells from Hakkaart et al., 2005	AOX	*C. intestinalis*	CIV dysfunction. shRNA targeted against COX10			[92]
COX-defective fibroblasts	AOX	*C. intestinalis*	CIV dysfunction. Deleterious COX15 gene mutation	Reduction in the superoxide production in COX15^–^ cells in the presence of antimycin	Hypertrophic cardiomyopathy	[92]
HEK293 Flp-In cells	AOX	*C. intestinalis*			Alzheimer’s disease	[94]
Complex I defective fibroblasts	NDH-2	*Arabidopsis thaliana*	CI dysfunction. CI-defective fibroblasts	The normalization of SOD activity		[106]
*Drosophila*						
w^1118^ *Drosophila*	AOX	*C. intestinalis*	CIV dysfunctions. Partial knockdown of COXVIc and complex IV assembly factor Surf1		Leigh syndrome	[95]
w^1118^ *Drosophila*	AOX	*C. intestinalis*		Reduction in the ROS production	Parkinson’s disease	[95]
w^1118^ *Drosophila* with knockdown of different complex IV subunits	AOX	*C. intestinalis*	CIV dysfunctions. Knockdown of different CIV subunits			
w^1118^ *Drosophila*	AOX	*C. intestinalis*		AOX abrogates induction of oxidative stress markers in a Drosophila AD model	Alzheimer’s disease	[94]
w^1118^ *Drosophila*	Ndi1	*S. cerevisiae*	CI dysfunction. Rotenone-induced inhibition, paraquat-induced inhibition	Mitigation of mitochondrial ROS production, oxidative damage		[105]
w^1118^ *Drosophila*	Ndi1	*S. cerevisiae*	CI dysfunction. Rotenone-induced inhibition	*Ndi1* expression in neurons, reducing ROS levels		[102]
*UAS-dCIA30 Drosophila*	*Ndi1*	*S. cerevisiae*	CI dysfunction. Reduced expression of CI assembly factor			[103]
w^1118^ *Drosophila*	*Ndi1*	*Saccharomyces cerevisiae*	CI dysfunction. Rotenone-induced inhibition	Reduction in whole tissue ROS levels		[104]
*Drosophila* lines 24,861 and 24,871	Ndx	*C. intestinalis*		Increased resistance to menadione-induced ROS production		[100]
Mice						
Mice (strain not specified)	AOX	*C. intestinalis*	CIV dysfunction. Cyanide-induced inhibition	Decrease in antimycin- and cyanide-induced superoxide overproduction		[93]
AOX^Rosa26^ mice, C57Bl/6J strain background	AOX	*C. intestinalis*	CIV dysfunction. Cyanide-induced inhibition Azide-induced inhibition. CIII dysfunction. Antimicyn-induced inhibition	Decrease in H_2_O_2_ production in succinate-supported mitochondria		[99]
AOX^Rosa26^ mice, C57Bl/6J strain background	AOX	*C. intestinalis*	CII dysfunction. Cigarette smoke condensate-induced inhibition. CIV dysfunction. Cigarette smoke condensate-induced inhibition	Decreases superoxide production		[96]
(cIII)-deficient Bcs1l^p.S78G^ knock-in mice AOX backcross with transgenic mice	AOX	*C. intestinalis*	CIII dysfunction. (CIII)-deficient Bcs1l^p.S78G^ knock-in mice		Lethal mitochondrial cardiomyopathy	[98]

## Data Availability

Not applicable.

## Supplementary Materials

The following supporting information can be downloaded at: https://www.mdpi.com/article/10.3390/ijms242216491/s1
