# Supplementary material for: Noncoupled Mitochondrial Respiration as Therapeutic Approach for the Treatment of Metabolic Diseases: Focus on Transgenic Animal Models
Source: Int J Mol Sci. 2023 Nov 18;24(22):16491. doi: 10.3390/ijms242216491 (PMC10671337; doi:10.3390/ijms242216491)
Supplement: Supplementary file 1 [file ijms-24-16491-s001.zip › ijms-2683384-supplementary.pdf]

Uncoupler	Stucture	Properties	Reference
**2,4-DNP**<br>(2,4-Dinitrophenol)	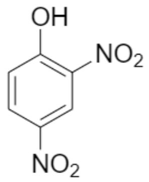	It lower the mitochondrial membrane potential, which abolishes overt reactive oxygen species (ROS) production and subsequently closes the uniporter involved in calcium influx	[55]
**FCCP**<br>(Carbonyl cyanide p-(tri-fluromethoxy)phenyl-hydrazone)	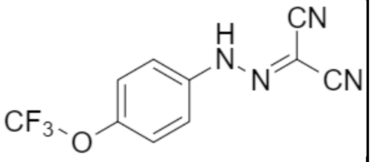	A potent mitochondrial uncoupler that inhibits oxidative phosphorylation by dissipating the proton gradient across the inner mitochondrial membrane, and as a result blocks mitochondrial ATP synthesis. Activate proton conductance through the plasma membrane and can influence a range of membrane proteins and ion channels that regulate membrane potential and cell volume.	[31]
**CCCP**<br>(Carbonyl cyanide m-chlorophenyl hydrazone)	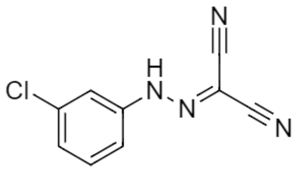	Inhibits oxidative phosphorylation by collapsing the proton gradient. One molecule of CCCP can transport many protons across the inner membrane, making it a potent uncoupler.	[134]
**BAM15** (N5,N6-bis(2-Fluorophenyl)[1,2,5]oxadiazolo [3,4-b]pyrazine-5,6-diamine)	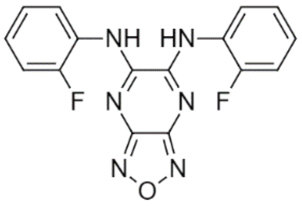	BAM15 decreases hepatic fat, decreases inflammatory lipids, and has strong antioxidant effects	[119]
**Niclosamide** (5-Chloro-N-(2-chloro-4-nitrophenyl)-2-hydroxybenzamide)	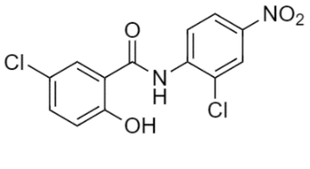	Potent activator of PINK1 by mitochondrial depolarization. Targeted to Wnt/b-catenin, mTOR and JAK/STAT3 signaling pathways	[40]
**Nitazoxanide** (2-(Acetyloxy)-N-(5-nitro-2-thiazolyl) benzamide)	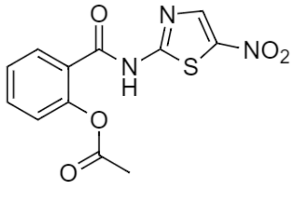	Mild uncoupling effect prevent MPP+ toxicity. Conferred significant protection against the loss of tyrosine hydroxylase (TH)-positive neurons of substantia nigra	[120]

**Oxyclozanide** (2,3,5-Trichloro-N-(3,5-dichloro-2-hydroxyphenyl)-6-hydroxybenzamide)	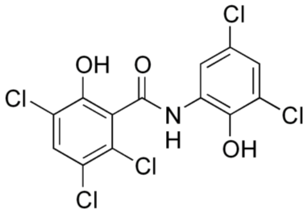	Mitochondrial uncoupling promotes pyruvate influx to mitochondria and reduces various anabolic pathway activities, inhibits cell proliferation and reduces clonogenicity of cultured colon cancer cells	[118]
**SR4** (1,3-Bis(3,5-dichlorophenyl) urea)	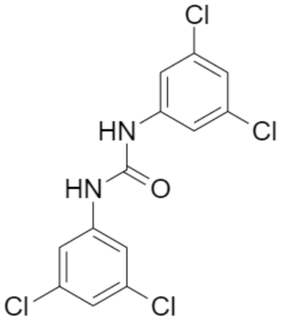	Mitochondrial uncoupler caused increase in mitochondrial respiration and dissipation of mitochondrial membrane potential. The reduction of cellular ATP production, activation of the energy-sensing enzyme AMPK, and inhibition of acetyl-CoA carboxylase and mTOR pathways, leading to cell cycle arrest and apoptosis HepG2 cells	[44]
**FR58P1a** (Dihydroxy-4,8,8-trimethyl-5-oxo-1,4,5,8-tetrahydroanthracene-1-yl)methyl 6-bromohexanoate)	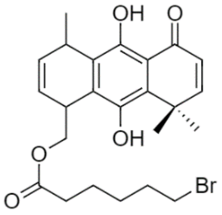	does not depolarize the plasma membrane and its effect on the mitochondrial membrane potential and bioenergetics is moderate suggesting a mild uncoupling of OXPHOS. activates AMPK in a Sirt1-dependent fashion.	[32]
**FH535** (2,5-Dichloro-N-(2-methyl-4-nitrophenyl) benzenesulfonamide)	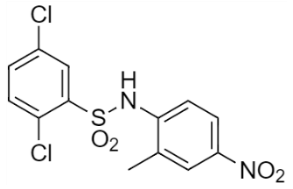	Mitochondrial proton uncouplers that independently activate AMPK and concomitantly inhibit Wnt signaling.	[45]
**F16** 3-[2-(1-Methylpyridin-1-ium-4-yl) ethenyl]-1H-indole	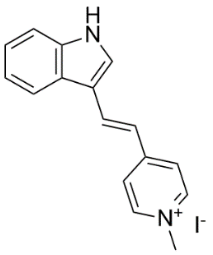	showed strong uncoupling activity in mitochondria, decreased intracellular ATP levels, decreased $\Delta\psi m$, and the release of cytochrome c, suggesting mPTP-mediated cytotoxicity	[136]

**Nemorosone**<br>(1R,5R,7S)-1-Benzoyl-4-hydroxy-8,8- dimethyl-3,5,7-tris(3-methyl-2-buten-1-yl)bicyclo[3.3.1]non-3-ene-2,9-dione	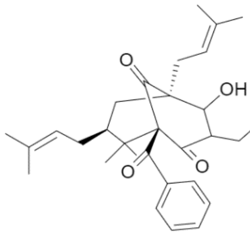	Induce a reduction in mitochondrial membrane potential (Δψm) with concomitant Ca2+ release in pancreatic cancer cells	[126]
**Guttiferone A**<br>(1R,5R,7R,8S)-3-(3,4-Dihydroxybenzoyl)-4-hydroxy-8-methyl-1,5,7-tris(3-methyl-2-buten-1-yl)-8-(4-methyl-3-penten-1-yl) bicyclo[3.3.1]non-3-en-2,9-dione	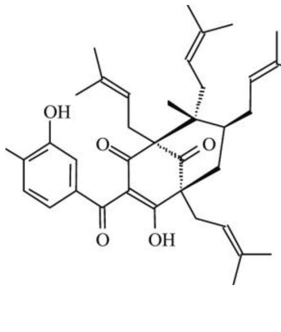	promoted membrane fluidity increase, membrane permeabilization, uncoupling (membrane potential dissipation/state 4 respiration rate increase), Ca^{2+} efflux, ATP depletion, NAD(P)H depletion/oxidation.	[130]
**CTU**<br>16-(([4-Chloro-3-(trifluoromethyl) phenyl] carbamoyl)amino)hexadecanoic acid	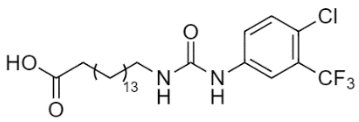	induced depolarisation of mitochondrial membrane, increased caspase-3/7 activity, and depleted ATP levels	[131]
**VE-3N**<br>formyl-2-methyl-4-(3-nitrophenyl)-1,4-dihydrpyridine-3-carboxylate	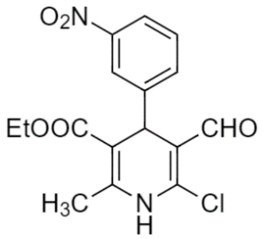	Dissipation of Djm and ATP production, suppression of respiration rate, reduction in ATP levels and Ca2+ uptake, induction of Ca2+ release, and increase in mitochondrial membrane fluidity and mitochondrial swelling in HepG2 cells	[137]
**Triclosan**<br>(5-Chloro-2-(2,4-dichlorophenoxy)phenol)	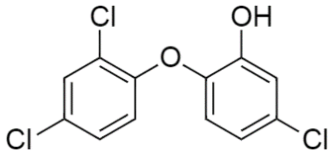	Significant reduction of mitochondrial membrane potential. This reduction did not appear to be accompanied by a corresponding decrease in mitochondrial mass, which may be indicative of weak uncoupling activity. stimulate mitochondrial turnover in a PINK-1-dependent manner.	[41]
**Sertraline**<br>(1S,4S)-4-(3,4-dichlorophenyl)-N-methyl-1,2,3,4-tetrahydronaphthalen-1-amine	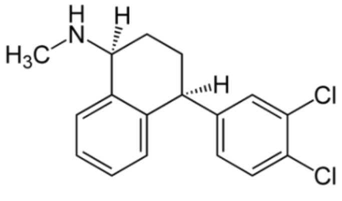	Significant reduction of mitochondrial membrane potential. This reduction did not appear to be accompanied by a corresponding decrease in mitochondrial mass, which may be indicative of weak uncoupling activity. stimulate	[41]

		mitochondrial turnover in a PINK-1-dependent manner.	
**Fisetin** (2-(3,4-Dihydroxyphenyl)-3,7-dihydroxy-4H-chromen-4-one)	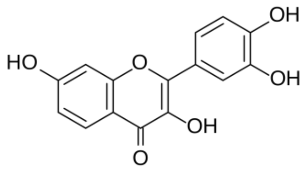	Can dissipate mitochondrial membrane potential (Ψm) by functioning as mitochondrial uncouplers. Prevention of neurodegeneration in aged *C. elegans*	[38]
**Quercetin** (2-(3,4-dihydroxyphenyl)-3,5,7-trihydroxychromen-4-one)	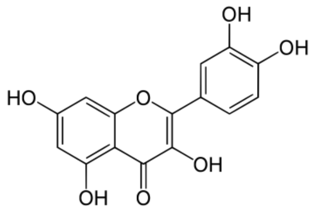	Can dissipate mitochondrial membrane potential (Ψm) by functioning as mitochondrial uncouplers. Prevention of neurodegeneration in aged *C. elegans*	[38]
**Apigenin** (5,7-Dihydroxy-2-(4-hydroxyphenyl)-4H-chromen-4-one)	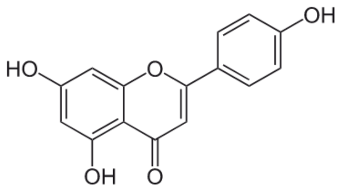	Can dissipate mitochondrial membrane potential (Ψm) by functioning as mitochondrial uncouplers. Prevention of neurodegeneration in aged *C. elegans*	[38]
**Chrysin** (5,7-dihydroxy-2-phenylchromen-4-one)	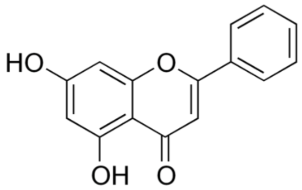	Can dissipate mitochondrial membrane potential (Ψm) by functioning as mitochondrial uncouplers. Prevention of neurodegeneration in aged *C. elegans*	[38]
**Catechin** (2-(3,4-dihydroxyphenyl)-3,4-dihydro-2H-chromene-3,5,7-triol)	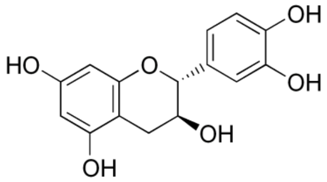	Can dissipate mitochondrial membrane potential (Ψm) by functioning as mitochondrial uncouplers. Prevention of neurodegeneration in aged *C. elegans*	[38]
**Naringenin** (5,7-dihydroxy-2-(4-hydroxyphenyl)-2,3-dihydrochromen-4-one)	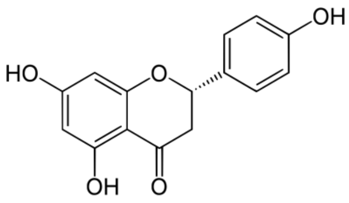	Can dissipate mitochondrial membrane potential (Ψm) by functioning as mitochondrial uncouplers. Prevention of neurodegeneration in aged *C. elegans*	[38]
**Bupivacaine** (1-butyl-N-(2,6-dimethylphenyl)piperidine-2-carboxamide)	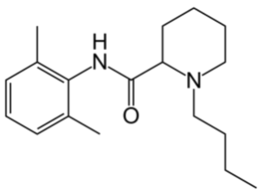	local anesthetic that is a validated mitochondrial protonophore	[122]

**TTFB** (4,5,6,7-tetrachloro-2-(trifluoromethyl)-1H-benzimidazole)	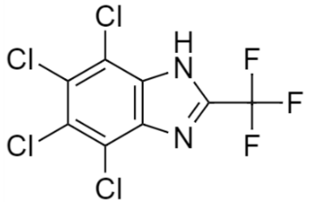	Herbicidal use. Due to mammalian toxicity and an uncoupling preference for brain mitochondria over liver mitochondria,has not been pursued as a therapy for metabolic or neurodegenerative diseases	[127]
**C4R1** (C4R1Rhodamine 19 butyl ester)	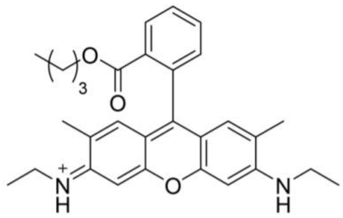	Cationic protonophore that is selective for mitochondria whose activity is dependent on membrane potential. Offers neuroprotection by reducing brain swelling and cognitive decline in a stroke model	[36]
**Ellipticine** (5,11-dimethyl-6H-pyrido[4,3-b]carbazole)	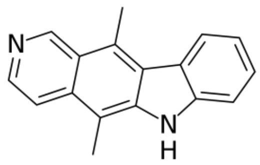	Alkaloid, topoisomerase II-β inhibitor that also has mitochondrial uncoupling activity. Cytotoxic, there are no current reports of medical use	[125]
**Salicylanilide** 2-hydroxy-N-phenylbenzamide	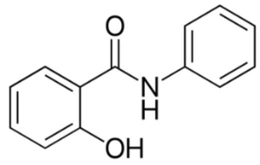	Mitochondrial uncoupling activity. The toxicity prevents its use as a therapeutic	[135]
**Tyrphostin A9/SF-6847** [(3,5-di-tert-Butyl-4-hydroxyphenyl)methylidene]propanedinitrile	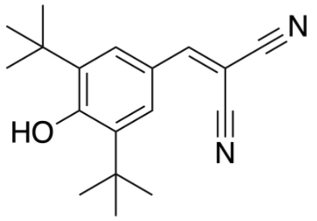	Most powerful uncoupler (over 1800x more potent that of 2,4-dnp). Low concentrations have been shown to be protective against glutamate-induced oxidative damage in neuronal cells	[133]
**CZ5**	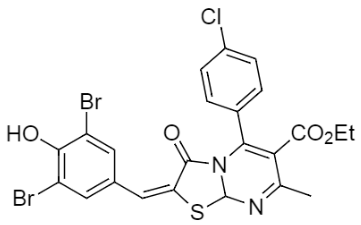	Promoted uncoupled respiration, improved glucose and lipid metabolism. May have therapeutic potential for treating high-fat diet-induced metabolic diseases	[123]
**C12TPP** dodecyltriphenylphosphonium	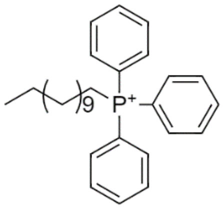	Uncoupling activity mediated by the recycling of endogenous fatty acids, which are natural uncoupling agents in the mitochondrial membrane. Suppress appetite, decrease body weight and fat mass without noticeable toxicity	[32]

**Usnic acid**<br>2,6-diacetyl-7,9-dihydroxy-8,9b-dimethyldibenzofuran-1,3-dione	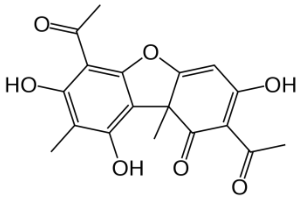	Directly inhibit and uncouple oxidative phosphorylation. Has a strong hepatotoxic agent that triggers oxidative stress and disrupts the normal metabolic processes of cells	[39]
**CDE**<br>4β-cinnamoyloxy,1β,3α-dihydroxyeudesm-7,8-ene	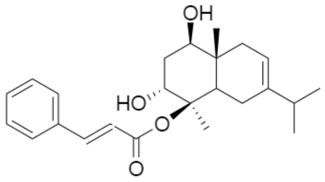	Natural mild uncoupler in liver mitochondria. Protonophore effect and membrane potential (Δψ) collapse. Largely used in folk medicines, potentially effective anti-obesity drug	[35]
**Sorafenib**<br>4-[4-[[4-chloro-3-(trifluoromethyl)phenyl]carbamoylamino]phenoxy]-N-methylpyridine-2-carboxamid**e**	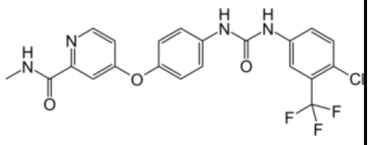	Hepatic mitochondrial uncoupler. Activator of AMPK and subsequent suppressor of mTOR. Clinical significance of low-dose sorafenib treatment for NASH/HCC	[43]
**Curcumin**<br>(1E,6E)-1,7-bis(4-hydroxy-3-methoxyphenyl)hepta-1,6-diene-3,5-dione	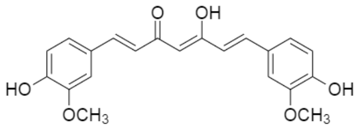	Displays all the characteristics typical of classical uncouplers. Causes a decrease in ATP biosynthesis in rat liver mitochondria, change in ATP:AMP ratio for activation of AMPK and its downstream mTOR and STAT-3 signaling	[42]
**SkQ1**<br>10-(6'-Plastoquinonyl)decyltriphenylphosphonium	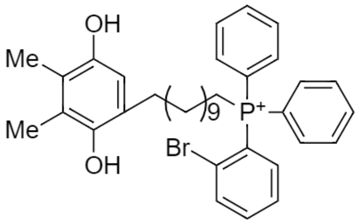	Inhibition of ROS at the site of their formation, and in two different ways:<br>• direct neutralization of ROS due to the oxidation of plastoquinone;<br>• decrease in the potential of the mitochondrial membrane.<br>Show favorable therapeutic effects on agerelated diseases such as heart infarction, heart arrhythmia, stroke, kidney ischemia, some kinds of cancer, eye pathologies (cataract, retinopathies, glaucoma, and uveitis), and osteoporosis	[47]
**C12R1**<br>dodecylrhodamine 19	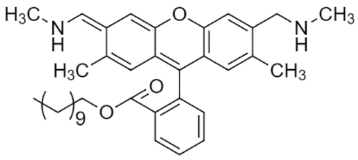	Penetrating cation from SkQ family has uncoupling properties, this fact making it highly potent for use in	[34]

		prevention of pathologies associated with oxidative stress induced by mitochondrial hyperpolarization.	
***ortho*-Carborane**<br>1,2-Dicarbadodecaborane(12)	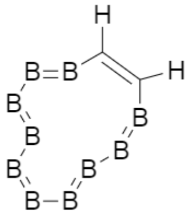	Stimulated mitochondrial respiration and decreased the membrane potential.<br>The applications of *ortho*-carborane and derivatives in the field of antitumor research mainly include boron neutron capture therapy (BNCT), as BNCT/photodynamic therapy dual sensitizers, and as anticancer ligands.	[121,132]
**DNPME**<br>2,4-dinitrophenol methyl ether	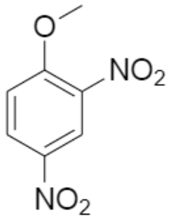	Liver-targeted DNP derivative that would be preferentially metabolized by liver and converted to DNP. DNPME both prevented and reversed nonalcoholic fatty liver disease, insulin resistance, and hyperglycemia in rat models of NAFLD and T2D without hepatic or renal toxicity.	[49]
**ES9**<br>Endosidin9	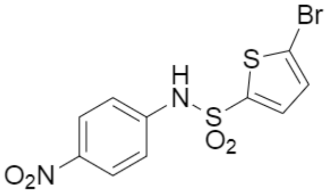	Mitochondrial uncoupler which is a potent inhibitor of CME in different systems and induces inhibition of CME not because of it`s effect on cellular ATP, but rather due to it`s protonophore activity that leads to cytoplasm acidification.	[138]
**Salsalate**<br>2-(2-hydroxybenzoyl)oxybenzoic acid	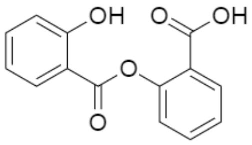	Strengthened expression of genes in mitochondrial uncoupling and mitochondrial electron transport. Also it decreased lipid accumulation in liver and epididymal white adipose tissue (eWAT), inhibited hepatic gluconeogenesis and improved insulin signaling transduction in eWAT. In addition, salsalate increased the expression of genes related to glucose and fatty acid transport and oxidation in skeletal muscle	[129]

**Valinomycin**<br>(3S,6S,9R,12R,15S,18S,21R,24R,27S,30S,33R,36R)-6,18,30-trimethyl-3,9,12,15,21,24,27,33,36-nona(propan-2-yl)-1,7,13,19,25,31-hexaoxa-4,10,16,22,28,34-hexazacyclohexatriacontane-2,5,8,11,14,17,20,23,26,29,32,35-dodecone	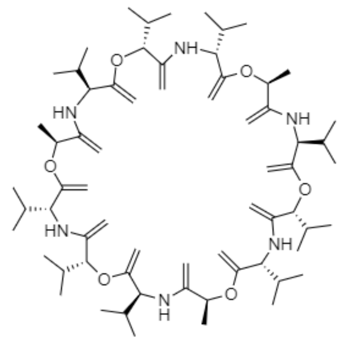	Act as a specific potassium ionophore leading to alkalinisation of the mitochondrial matrix. This effect on matrix pH can influence the dissimilar modulation of ROS levels.	[124]
**5BMF**<br>6-(bromomethyl)-3',6'-dihydroxyspiro[2-benzofuran-3,9'-xanthene]-1-one	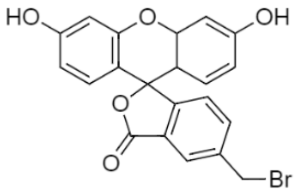	Selective accumulation in mitochondria leading to its swelling, increased ROS production, and outer membrane rupturing which inducing the death of cancer cells	[7]
**T3**<br>(2S)-2-amino-3-[4-(4-hydroxy-3-iodophenoxy)-3,5-diiodophenyl]propanoic acid	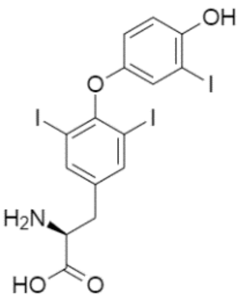	Stimulates thermogenesis by inducing metabolic inefficiency through the induction of the mitochondrial uncoupling protein, UCP1.	[46]
**OPC-163493**<br>5-{5-methyl-2-[4-(trifluoromethyl)phenyl]thiazol-4-yl}-3H-[1,2,3]triazole-4-carbonitrile	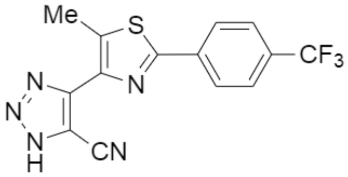	OPC-163493 is a protonophore with milder activity than carbonyl FCCP.<br>It may encourage cells to use the most efficient catabolic (respiratory) mechanisms they can and promote optimal usage of available energy sources within the cells, to maintain Δψ.	[128]
